# Companion animals support human wellbeing and health: evolutionary, behavioral and physiological contexts

**DOI:** 10.3389/fpsyg.2026.1800617

**Published:** 2026-06-01

**Authors:** Kurt Kotrschal

**Affiliations:** Department of Behavioral Biology, University of Vienna, Vienna, Austria

**Keywords:** animal-assisted pedagogy, animal-assisted therapy, animal-assisted activities, biophilia, dog, domestication, human-animal relations, social homoeostasis

## Abstract

This review deals with the questions why humans want to live with other animals and how this is at all possible. In fact, it has become common sense that living and working with companion animals entails major benefits for human wellbeing and health, albeit with the caveat that the positive experience of private keepers and practitioners in pedagogy and therapy is not always backed by scientific scrutiny. The present focus is on relevant aspects of the “Darwinian continuum,” which provides humans and other animals with a shared social toolbox, including brain, physiology and behavioral organization. It is discussed why domesticated animals are particularly suitable companions, why dogs are a special case, and why anthropomorphizing other animals may be as much an asset as a burden. In fact, living with companion animals is a human universal, which seems basically motivated by biophilia (the human-typical interest in nature and animals), and by striving for social homoeostasis (for social contexts supporting wellbeing and health). Between-species socializing is possible because of common phylogeny and functional convergence, resulting in matching social mindsets and behavioral systems. It is based on shared principles of behavioral organization, of thinking and decision making, on shared neuronal, physiological and psychological mechanisms, on virtually identical basic affects, and on the shared stress and calming systems. Finally, the social toolbox shared between humans and other animals also suggests a relatively moderate socio-cognitive gap between humans and other animals.

## 1 Potential benefits of private animal companionship as well as animal-assisted activities and interventions

### 1.1 Why humans are needy of other animals and nature

The focus of this review is on the comprehensive social toolbox shared between humans and other animals due of common phylogeny and parallel evolution. Such common ground may contribute to explain why we can engage in between-species social relationships. The general tendency of humans to associate with other animals seems a “human universal” (Brown, 1991; Christakis, 2019), related to a peculiar human nature. In fact, humans evolved into the vertebrate top socio-cognitive specialists (Kotrschal, 2019; Bennett, 2021), with the relatively largest brains, containing the most neurons (von Bartheld et al., 2016). This is not just a blessing. The ability to reflect and conceptualize comes with an obligation of responsibility; also, brain evolution constrains other functional domains, such as digestion and reproduction (Tsuboi et al., 2014; Lesch et al., 2022). And for proper functioning, the high-tuned human brain is particularly needy of optimal social conditions during development and throughout life (Kotrschal, 2019; van IJzendoorn et al., 1995). This can be conceptualized as “social homoeostasis” (Matthews and Tye, 2019). For optimal individual development and functioning in the social domain (i.e., in the sense of well developed “executive functions”; Diamond, 2013) and generally, for getting ones dispositions on the ground, the central factor is reliable and sensitive caregiving early on (Bowlby, 1969/1982, 1979; Hinde and Stevenson-Hinde, 1990). But in addition, growing up in contact with nature and animals seems a mandatory support (Louv, 2005) of the emotional, social and cognitive development of children and the wellbeing, health and mental resilience of adults (Headey et al., 2008). In fact, the available evidence indicates (Julius et al., 2012) that *Homo sapiens* is adapted to live with other animals, which seems to be the human default condition (Kotrschal, 2024).

### 1.2 How companion animals may benefit quality of life and health

Living on good terms with companion animals (preferred here over “pet” because it emphasizes animals as social partners) may enhance the human quality of life in many ways. Living with animal companions particularly touches the social, wellbeing, physical as well as mental health aspects of the multifaceted “quality of life” construct (Theofilou, 2013)—much in the sense of One Health (Pitt and Gunn, 2024) and One Welfare (Tarazona et al., 2020).

Companion animals support a wide range of professional interventions, pedagogic and therapeutic. This will be summarized in the following. The main text will focus on structures, patterns, mechanisms and functions (in the sense of Tinbergen, 1963) contributing to explain why human-animal partnership is desired, possible, and how it potentially supports wellbeing, health and longevity—ideally for both, the human and the animal partners (Barcelos et al., 2023; Capela e Silva et al., 2024; Kotrschal, 2024; O’Haire, 2010). Most suitable companions are domesticated animals, who are genetically adapted to live with humans and are therefore, preferable over tamed wildlife for practical and ethical reasons (IAHAIO declarations)^1^. For practical reasons and because of their social compatibility (below), dogs are the most important supporters of private owners and in professional applications as well. Therefore, most of the relevant literature this review is on human-dog interactions.

Interactions with animal companions may activate the calming system (DeVries et al., 2003), decreasing owner resting heart rate, blood pressure and triglycerides levels (Beetz et al., 2012; Fine et al., 2019; Friedmann et al., 2011). This can be explained by physical, mental and social contributions: Most dog owners engage in more physical activity than comparable non-owners; interacting with companion animals may lower cortisol and increase oxytocin, serotonin, and dopamine, fostering feelings of calm and happiness. Thereby, symptoms of depression and anxiety may be alleviated, the more as owners may experience unconditional love by their animal companions without feeling judged. The daily routines of feeding, grooming, and exercising a pet provide structure and a sense of accomplishment, which is particularly important for the physical and cognitive wellbeing of seniors (Applebaum et al., 2023).

Phylogenetically, humans are socio-cognitive specialists (Herculano-Houzel, 2016), implying, that their cognitive potential, wellbeing, health and even survival depends on an adequate socially environment (Bowlby, 1979; Kotrschal, 2019; Matthews and Tye, 2019). No wonder that the benefits listed above are substantially supported by the social connectivity, a companion animal may provide. Dogs in particular, are “social lubricants,” facilitating contact with neighbors and strangers and thereby, expanding social networks, reducing isolation and combatting loneliness. In fact, companion animals have a considerable potential of satisfying basic human needs, such as touch, emotional connection, purpose and efficacy (Kanat-Maymon et al., 2016).

Children enjoy developmental benefits regarding empathy, responsibility and social skills by caring for, and living with, companion animals (Melson, 2003). Animal companionship—under the close and informed surveillance of adults—is particularly indicated for children with developmental challenges like ADHD or autism (Wright et al., 2015). Given the psychosomatic linkage and also, the beneficial effects for a diverse microbiome, exposure to companion animals such as cats and dogs, particularly in early childhood, can strengthen the immune system and reduce the long-term risk of developing allergies and asthma (Salas Garcia et al., 2019).

### 1.3 Animal assistants in pedagogy and therapies

Such positive effects of companion animals suggest that professional interventions also may benefit from animal assistance in animal-assisted activities (AAI), animal-assisted pedagogy (AAP) or animal-assisted therapy (AAT). A major difference would be that the effectiveness of privately kept companion animals is generally attributed to the quality of the particular long-term dyadic relationships (Cimarelli et al., 2021; Hardie et al., 2023; Schöberl et al., 2012); this is necessarily much less the case between clients and animal assistants of therapists or pedagogues. Hence, some factors making animal assistance effective are seemingly unrelated to social closeness and bonding. Major candidates are biophilia and social homoeostasis (below), intrinsic to humans and, to some extent, probably also to companion animals. Such common grounds will be the main topic of this contribution.

In animal-assisted activities (AAA), pedagogy (AAP) and therapy (AAT) animals are active agents, generally in structured interactions by respective professionals (e.g., Fine, 2019; Fine et al., 2025). Assistant animals provide comfort, trust and motivation for clients to participate in the program, act as social facilitators toward clients and/or as “metaphors” for a patient’s own life. Generally, animal assistance increases treatment efficiency by triggering positive biological mechanisms (Beetz, 2017; Wagner et al., 2022).

Animals function as “social lubricants,” decreasing initial resistance to therapy, particularly in children (Fine and Weaver, 2018). A therapist with an animal is generally perceived as more approachable and trustworthy, making clients more ready for opening up, the more as animals provide immediate, non-judgmental responses to clients (Kotrschal, 2014). For example, a horse, chicken or dog might move away if it senses a person’s unbalanced mood, thereby providing feedback to the clients to recognize and regulate their emotions in order to establish contact. The general human inclination (Urquiza-Haas and Kotrschal, 2015, 2022) to project feelings, attitudes, etc., onto animals, also applies to clients, providing the therapist with a tool to work with. Finally, animals offer a sense of acceptance free of the kinds of social judgment, often burdening interactions between humans. This may be particularly relevant in clients in the aftermath trauma or abuse.

Connected to facilitating such psychological and physical effects, interacting with animal—mostly dogs, but also various farm animals—in a variety of professional settings may trigger measurable bio-physiological changes supporting recovery (Fine, 2019). For example, mere petting or making eye contact with an animal increases the release of oxytocin, dopamine, and serotonin, improving mood and promoting joy and trust (Beetz et al., 2012; Grahn et al., 2021; Julius et al., 2012). As oxytocin is an effective antagonist of the stress hormone cortisol (DeVries et al., 2003), such interactions have calming effects, coming also with reduced blood pressure and heart rate. Hence, interacting with companion and assistant animals supports social homoeostasis (van Hooff and Aureli, 2014; Allsop et al., 2025) on the side of the humans and, potentially, also on the side of the animals. Furthermore, interacting with animals also has the potential of reducing the perceived intensity of pain, in cases even allowing for reduced pain medication (Beetz et al., 2012).

Animal assistance activates motivational resources in the clients and potentially—also in the professional human partners (Julius et al., 2012), which is particularly important in physical and occupational therapy settings. By grooming a horse or throwing a ball for a dog, clients playfully practice motor skills at different levels. And when animals are involved, clients are more willing to engage in physical activities, or participating in exercises (Kovács et al., 2024). Not the least, caring for an animal introduces structure, routine and responsibility, for example, helping patients with dementia to re-activate their emotionality, speech and memory, or ADHD clients to improve their focus (etc., Hoagwood et al., 2017).

The kind and species of assistant animal employed depends on utility and indication. Most common are dogs, which are humans’ most widely distributed and also historically closest animal companions (Kotrschal, 2023, 2024). They assist in a wide variety of training and treatment settings, for example, social skill training, anxiety reduction, as motivators in speech therapy, or in practicing to read and calculate, etc (Fine, 2019). The keeping and handling of horses is considerably more costly and more effort, but they may be efficient in training motor and physical balance (e.g., for patients with spastic symptoms), in ergotherapy, and in regulation emotions, etc. Farm animals, such as cattle or goats may be valuable partners in many practical pedagogic and therapeutic applications (e.g., Malinowski et al., 2018). Chickens, rabbits, guinea pigs or other small animals may be ideal for sedentary patients or those with sensory needs. However, due to their convenience, small animals are particularly easily exploited. Hence, as the case in all animal companions and assistants, in-depth knowledge of their needs is mandatory to avoid their exploitation—for ethical reasons and to ensure their active and positive contributions.

### 1.4 How good is the evidence?

Scientific scrutiny only ambiguously supports the positive experience of private keepers and of practitioners in pedagogy and therapy and demands critical distance to the many positive results reported (above). In fact, much of the scientific literature remains correlational or otherwise confounded (McNicholas et al., 2005; Herzog, 2011). For example, reviews on loneliness do not reveal a clear protective effects of companion animals (Gilbey and Tani, 2015; Kretzler et al., 2022; Moshfeghinia et al., 2025). Also, longitudinal analyses failed to report beneficial associations for a range of mental health measures (Parsons et al., 2024; Sharpley et al., 2020; Hansen et al., 2024). And although meta-analyses linked dog ownership with decreased mortality, this observational evidence is still subject to confounding and concerns about selection criteria, e.g., regarding mortality causes (Kramer et al., 2019; Bauman et al., 2020).

The introduction (above) itself hints at some reasons for such negative or problematic results: Everyday animal companionship mingles with interactions and formal animal-assisted interventions, differing in mechanisms, evidence bases, and validity. Also, there is a temptation to generalize evidence from one these domains to the others, blurring inferential boundaries. The validity of general statements on the positive effects of human-animal companionship and reviews as well as of the meta-analyses thereof, are generally constrained by an enormous heterogeneity in intervention format, dosage, populations, comparators, and outcome parameters. In fact, weak scientific standards usually necessitate the exclusion of majority of contributions found by systematic literature searches from reviews. For example, our intended meta-analysis of dog-assistance in special education turned into a qualitative review (Meixner and Kotrschal, 2022), because only 18 of the 822 articles found via a systematic literature search could be integrated (!); the great majority did not meet even them most elementary scientific standards and the 18 remaining publications were still very heterogenous in scope, settings and outcome parameters.

Hence, the general overview above is intended as a frame for the central neuro-behavioral topic of this review. Notwithstanding whether and how companion animals and animal assistance works in pedagogy and therapies, this became such an ubiquitous grassroot movement that the evolutionary, and bio-psychological context for this success story demands attention. And there are also (well known) caveats toward an all-too critical view of the field. Above all, negative results are weaker than positive ones, not the least because they can never be considered as a proof of non-existence. Also, the often sweepingly wide scope of reviews mentioned above, focusing on “pet owners” in general and on populations rather individuals may cancel out positive effects potentially found in sub-samples (pooling-fallacy). For example, in an early study on the social effects of dogs in a class (Kotrschal and Ortbauer, 2003), very different, socially positive effects of the dog present were found in the different, children. An analysis at the population level would have canceled out these effects and would have resulted in negative results.

## 2 Where the urge of *Homo sapiens* to be social with other animals comes from

As humans across cultures have always lived with companion animals (Serpell, 1986), this lifestyle qualifies as a “human universal” (Brown, 1991; Christakis, 2019; Kotrschal, 2019). Still, it is not self-evident why in an urbanized world, the keeping of cats and dogs is even increasing and seems to be increasingly important to their human partners^2^. How “truly social” are such relationships between different species, how are they achieved and maintained? Finally, how come, that companion animals may support wellbeing, health and longevity of their owners (and vice versa, but see above) and efficiently assist in a variety of pedagogic ant therapeutic settings? Barcelos et al. (2023) listed twelve non-mutually exclusive psychosocial hypotheses and constructs: (1) social catalyst-repellent, (2) emotional contagion and empathy, (3) social support, (4) biophilia, (5) attributed fault, (6) social norms, (7) annoyance by noises, (8) routine, (9) caring, (10) exercise, (11) learning, and (12) affective touch. Presently, the focus is on “biophilia” and “social homoeostasis,” as they seem to be the central, evolution-based, and hence, most embracing concepts, integrating most of the others.

### 2.1 Biophilia

The ubiquity of animal companionship over most human cultures suggests that pet keeping is rooted in a specifically human nature and mind (Kotrschal, 2019). Fromm (1981) and Wilson (1984) termed the human interest in nature and animals “Biophilia” (Kellert and Wilson, 1993). On the base of their large and powerful brain humans were capable to develop cultures and lifestyles, fit for colonizing most of the earth (Shipman, 2015), mainly by an evolved mental background featuring strong interest in nature and animals—which is still part of human nature (Kotrschal, 2019). Due to their “evolutionary esthetics,” it seems a human universal to prefer living in park-like, relatively open landscapes with water bodies, providing shelter as well as view into distant space (e.g., Thornhill, 2003; Wilson, 1984). Support of the biophilia hypothesis also comes from evidence, that babies independent of parental cultural background are born with a strong interest in animals (Wedl and Kotrschal, 2009; Kotrschal, 2014), as measured via the time babies spend being interested in pictures of, or real animals (DeLoache et al., 2011). This hints at the important roles of animals in the evolution of modern *Homo sapiens* and at their role for optimal child development (Kotrschal, 2016a; Melson et al., 2025; Reilly et al., 2024).

Although biophilia seems uniquely human, domestication has produced—to different degrees in different species—“homophilia” (i.e., being intrinsically interested in humans) in a variety of animals, disposing them as social companions of humans. In contrast to the ancestral wildlife, domesticated animals integrate humans into their specific “Umwelt” (in the sense of von Uexküll, 1909). For example, dogs are inherently more interested to interact with humans than equally raised wolves from early puppyhood on (Cimarelli et al., 2024; Gácsi et al., 2005; Range and Marshall-Pescini, 2022; Kotrschal, 2022).

### 2.2 Social homoeostasis

The concept of “social homoeostasis” (SH) integrates the bio-psychological, physiological and health-related domains of social behavior (Matthews and Tye, 2019) by considering the benefits it provides for social partners. In fact, the SH concept includes the structures, mechanisms and functions covered in this contribution. It is about the steady-state balance individual brains of highly social species, such as humans and their companion animals, try to maintain via adequate social contacts, regulating physiological and processes toward adaptive social optima (Kotrschal, 2013; Kotrschal et al., 2023). Socializing, including touch, etc., is not just emphatically and altruistically motivated, but before all, benefits the physiology, wellbeing and social standing of the contacter. Thereby, the SH concept emphasizes the benefits for both sides in a social interaction, including the partners in human-animal interactions. Matthews and Tye (2019) distinguish three functional components of SH: The sensory systems, the prefrontal cortex and hypothalamic brain control centers as well as the psychological, physiological and behavioral effectors; there is neural and humoral communication within the system, notably by dopamine, oxytocin cortisol, etc.,—which are mainly the messengers of the stress and calming systems (below; Grahn et al., 2021). Such, the SH system is not “just” a bio-psychological construct, but qualifies as an evolved (Tinbergen, 1951, 1963) behavioral system (e.g., Hinde and Stevenson-Hinde, 2006).

As generally true for all phenotypic traits, the setpoints of individual SH systems are adjusted by genetics, epigenetics as well as social experience (Jablonka and Lamb, 2014); this determines how individuals regulate their social behavior, which, in turn, feeds back on the epigenetics, affecting SH in the generations to come. In humans, the kind of caregiving received in the first years of life affects lifelong expectations toward social partners, affective regulation, openness to explore, resilience to mental conditions, etc (Bowlby, 1969/1982, 1979; Pietromonaco and Powers, 2015). Even in adulthood, social experience may modulate SH setpoints. For example, long-term social isolation may lead to antisocial attitudes, hypervigilance and sociophobia, all affecting wellbeing, health and life expectancy in a negative way (Brandt et al., 2022). This kind of context-dependent modulation is remarkable, particularly in the light of the present loneliness pandemia. Social companionship with animals can buffer against such effects, the more as dogs, for example, are considered social lubricants, improving the contacts with other people. Companion animals even have the potential, to buffer children against the most severe effects of dysfunctional social caregiving early on (Junça-Silva, 2025; Reilly et al., 2024).

### 2.3 Why *Homo sapiens* CAN be complexly social with other animals

The human desire to live with other animals in one form or another alone cannot explain how such relationships are achieved and maintained. What are the mechanistic substrates—motivational, neuronal and humoral—of socializing and decision making between different species? Actually, some of these mechanisms for enabling human-animal companionship discussed below may be more social-specific than others. For example, the attachment/caregiving behavioral system (Bowlby, 1979; Hinde and Stevenson-Hinde, 1990) would be specifically social, whereas the appetitive affective system (the “seeking” system; Panksepp, 1998, 2005) would be more general, providing drive over many behavioral domains, including social. Ultimately, all systems that keep organisms alive will also contribute to the social domain. In the following, some of the more specific mechanisms will be discussed.

#### 2.3.1 A shared social toolbox

Starting top down, any reasonably complex social behavior needs social mindsets hosting the motivational components (Bernard et al., 2005) for maintaining long-term individualized dyadic relationships. Such relationships are based on individual recognition and episodic social working memory, furthermore, on the ability to adjust to the social context, requiring awareness of one’s own position in the social web. Complex sociality is based on mechanisms to inhibit/control affective behavioral impulses, for delaying actions and for integrating experience into appropriate social behavior. In cooperative species, the range of shared features of the social mindset even includes inequity aversion (McGetrick and Range, 2018; Oberliessen and Kalenscher, 2019), i.e., the ability of recognizing when being “unfairly” treated. This requires the capability of forming mental representations on the relevant socio-ecological details, i.e., a social episodic memory (Clayton et al., 2001; Allen and Fortin, 2013). In sum, such a complex mental mindset needs to be based on a toolbox allowing for considerable context-specific behavioral flexibility.

“Behaving socially appropriate” needs the individual skills of adaptively responding to social context, ultimately (in the sense of Tinbergen, 1963), balancing one’s own interests with group functioning (Liu, 2024). In essence, it is about the regulation of distance and closeness to other group members, about establishing friendship, partnership and alliance—and also, of knowing thy foe. Within species-specific ranges, proximity seeking will depend on the quality of particular long-term dyadic relationships, determining the potentials of social support—including emotional (Kotrschal, 2013). In socially complex animals giving and accepting different-level social support is key for individual wellbeing, social functionality and ultimately, evolutionary fitness (Kotrschal et al., 2023). In fact, “emotional social support” interacts directly with the background of bio-psychological, physiological mechanisms (below).

In the following I will focus on the proximate and ontogenetic levels of behavior (Tinbergen, 1963) substantiating why people and their companion animals are indeed, able to engage in mutual social relationships, and why this is relevant for animal assisted activities (AAA). I will argue that this is possible because of a “social toolbox,” shared due to phylogeny as well as parallel evolution, including most of the components and mechanisms comprising social brains and physiologies (comp. “One Biology”: Tarazona et al., 2020).

Central components of this shared “social toolbox” include the common principles of how behavior is organized in the animal kingdom, which are particularly relevant for the (interspecies) communication of affects (below). Furthermore, this toolbox includes a range of brain mechanisms and functions, for example, the brainstem–diencephalic “social network” (Goodson, 2005), commanding instinctive socio-sexual behavior over the vertebrate kingdom; this network has remained virtually structurally and functionally unchanged since the Paleozoic (O’Connell and Hofmann, 2011). Other important toolbox elements are the conservatively preserved (i.e., evolutionary homologous) mechanisms of stress management and calming (Porges, 2009; DeVries et al., 2003; Grahn et al., 2021) and also, the basic affective systems (Panksepp, 1998, 2005) humans share with other animals. Particularly important tools in this box are the mechanisms how relatively stable individual temperaments and personalities develop between genome, epigenome, social and societal embedding (Jablonka and Lamb, 2014).

A majority of elements of the vertebrate social toolbox are shared between species because of their common phylogeny. For example, the basic elements of the conservative (below) social brain emerged early in phylogeny (Bennett, 2021, 2023). Hence, they are “homologous” in an evolutionary sense. In contrast, a striking example of parallel evolution (i.e., analogous development) would be the functionally identical mammalian prefrontal cortex and the bird *Nidopallium caudolaterale*, which are composed of identical neural elements, but are phylogenetically and ontogenetically assembled into very different structures (Güntürkün, 2005). One of the major selective pressures for this parallel evolution was probably the need to control for appropriate social behavior.

##### 2.3.1.1 Common principles of behavioral organization

Mutual understanding between humans and their animal companions is possible because major principles of behavioral organization are shared due to common phylogeny. For example, affects and emotions (i.e., unconscious or conscious, respectively) are communicated via mimics and body language (Ekman, 1999; Darwin, 1897; Eibl-Eibesfeldt, 1967). Such affective motor patterns are based on evolutionary prefabricated, and therefore, quick and easy-to-use behavioral elements (“action patterns”; Lorenz, 1978; Schleidt, 1974), residing in “pattern generators” (i.e., networks of spinal cord motor neurons; e.g., Frigon, 2012). Therefore, such instinctive behavioral components come relatively stereotypical and species-specific in shape, but may vary widely in intensity (Eibl-Eibesfeldt, 1967).

In contrast to the innateness of affective motor patterns, interpreting them within or even between species depends heavily on learning, though in combination with innate motivational components (i.e., specific interest; Marler, 2014). This explains why, for example, humans and dogs are able to learn to interpret each others’ affective expressions (Albuquerque and Resende, 2023). Although the Gestalt of affective mimics is very different in humans and dogs, the (instinctive) behavioral principles of expression are virtually identical—including the necessity of learning to interpret them. This creates a window of opportunity for communicating and relating between-species. Although there are seemingly sensitive periods for such learning in both, humans and dogs (Scott and Fuller, 1965; Woodard and Pollak, 2020), this is also a lifelong process, particularly in species with a learning-based behavioral organization.

##### 2.3.1.2 Vertebrate brain evolution as the basis for a common social mindset

The vertebrate brain evolved through a series of key innovations (Bennett, 2021, 2023; Kotrschal, 2023; Striedter and Northcutt, 2020). As a rule, ancient neural elements are conservatively kept over phylogeny and put into service in novel contexts when needed (Curley and Keverne, 2005). Some 600 million years ago, the bilaterian body plan emerged, necessitating the concentration of sensory systems and of a central nervous system (CNS) at the anterior pole. From very early on, this CNS assembled into the serial arrangement still characterizing modern vertebrate brains. From early on, these vertebrate brains needed to be capable of oriented steering and of providing appropriate behavioral responses to different environmental qualities. Systems for affective evaluation, stress management and association learning were needed, making it possible to expand the reflexive here-and-now responses over longer periods of time.

Some 450 million years ago, the development of “true” jaws from gill arches (DeLaurier, 2019) selected for an active, heterotrophic lifestyle; increasingly complex predator-prey systems selected for the expectation and curiosity, for trial-and-error learning, for remembering and recognizing (episodic) patterns and for acquiring and storing spatio-temporal maps—mainly via an associative forebrain extending the integration of olfactory stimuli into other sensory, affective and integrative domains. A capability of storing complex mental representations emerged (Bennett, 2021, 2023). All of these early developments have made it nearly unmodified into the brains of currently existing species. With the first mammals, some 220 million years ago, a Neocortex developed, composed of neural columns, which are basically general-input central processing units with a complex standard neural architecture. With this innovation a tool emerged for anticipating the immediate future. More brainpower is easily achieved by increasing the number of these neocortical columns.

Such a recap of 600 million years of brain evolution (Bennett, 2021, 2023; Striedter and Northcutt, 2020) makes clear why humans share major brain components with other animals. In fact, the basic social toolbox, the needs and midsets may be more similar between humans and other animals than many philosophers in the Aristotelian tradition (Russell, 1950; comp. Kotrschal, 2018, 2023) may have been, and are still, ready to acknowledge.

##### 2.3.1.3 The “conservative” social brain systems

One of the major coordination centers for mainly instinct-based social behavior is the so-called “vertebrate social behavior network” (Goodson, 2005) in the brain, consisting of seven diencephalic and brain stem nuclear areas. This social network remained essentially unchanged in structure, neurochemistry and functionality over 450 million years. In fact, it is the phylogenetically most conservative part of the vertebrate body (O’Connell and Hofmann, 2011). What had been adjusted over the course of social and cognitive evolution, were mainly the effector systems (Adkins-Regan, 2009). Similarly conservative are the thalamic and striatal systems of forebrain affectivity (Nesse et al., 2016), including relay structures for (socio)positive and fear/stress contexts, such as the caudate nucleus and the *Amygdala*.

In fact, MRT studies in dogs have revealed human-identical functioning of the caudate nucleus and other brain areas (Berns, 2013), also interacting with the closely linked behavioral systems of attachment and caregiving (Bowlby, 1979; Hinde and Stevenson-Hinde, 1990); the latter are essential for building and maintaining long-term relationships—both, within and between species (Julius et al., 2012). A core element of attachment and caregiving and of social homoeostasis in general (above) is the hypothalamic oxytocin (OT) system stimulated by socio-positive contexts, mediates calming (Grahn et al., 2021) and cooperation, as well as trust between close partners and within groups (Marsha et al., 2017; below). The setpoints of all these systems are profoundly modulated by early social experience (Bowlby, 1979; Zhang et al., 2022). Individual attachment patterns will lifelong affect social expectations and relationships with social partners (Shaver et al., 2014), including companion animals (Julius et al., 2012).

Due to the Darwinian phylogenetic continuum, humans share with other amniotic animals basic affective brain systems (above; Panksepp, 1998, 2005)]: seeking, rage, fear, lust, care, attachment, panic, and play, which are all motivators of their own behavioral contexts. For example, attachment and caregiving are a twin behavioral system (Bowlby, 1979; Hinde and Stevenson-Hinde, 1990) at the core social behavior of humans and probably, to some degree, of all big-brained mammals (Curley and Keverne, 2005); early social experience (i.e., the quality of caregiving) shapes the individual primary social mental representation (John Bowlby’s “internal working model”), affecting how individuals relate to their (social) environment later in life. This includes and affects the regulation of emotions and also, resilience against physical and mental problems, virtually “from the cradle to the grave” (Zhang et al., 2022), as John Bowlby put it. Particularly attachment and caregiving are key behavioral systems also in relating to companion animals (Julius et al., 2012).

These shared systems of affects and emotions are at the core of any social communication and relationships, within species, but also between humans and other animals (Ekman, 1999; Darwin, 1897); being able to read and understands the affective expressions of others (above; Bowlby, 1979; Singer, 2006) not the least is a necessary requirement for being capable to empathize within and between species, important in human-animal companionship and in AAI.

##### 2.3.1.4 The “modern” social brain

The phylogenetically old parts of the brain cross-talk with the modern, “cognitive” and associative components, the mammalian prefrontal cortex or the bird *Nidopallium caudolaterale* (above; Damasio and Damasio, 2012). These are the relay centers for allowing individuals to behave appropriately in social webs (Adolphs, 2003; De Waal, 1982), for conceptualization, unconscious and conscious decision making and thinking, as well as “inhibition” (Levita et al., 2014), i.e., the ability to control impulses. The quality of prefrontal cortex-based decision making is not hard-wired, but rather develops it’s individual ways of interacting with the environment between genetic and epigenetic heritage and social as well societal conditions (Jablonka and Lamb, 2014). Relevant estimates of individual prefrontal cortex functioning are provided by the psychological construct of “executive functions” (EF; Miyake and Friedman, 2012), integrating impulse control, the flexibility to adjust to environmental variation, social episodic memory (Amodio, 2019), goal-orientation, etc.

Even major mechanisms of social intelligence and thinking are shared with other animals. In fact, dogs are able to read human affective expressions (Müller et al., 2015; Range and Marshall-Pescini, 2022), show inequity avoidance (Range et al., 2009) and preferentially lead those human partners to boxes with toys or food that they have previously experienced as cooperative (Heberlein et al., 2016), and the previously non-cooperative humans to empty boxes (Heberlein et al., 2017). In fact, dogs—as humans—are capable of thinking and decision making based on mental representations (Topal et al., 2006), which also logically means that dogs navigate their behavior on the base of mental representations and some consciousness. This probably also applies to other social mammals and birds, as suggested by the “Darwinian continuum” and by increasing scientific evidence (Zentall, 2023).

##### 2.3.1.5 Social physiology

Stress systems evolved to cope with unfavorable conditions, physical, predators, competitors, etc. The substantial modulation of these physiological systems in social contexts reflects the general importance of social behavior at both, species and individual levels (Kotrschal, 2013; Kotrschal et al., 2023; Wascher et al., 2008). As the case with basal brain mechanisms (above), the major stress systems for coping with environmental challenge and variability were also conservatively maintained over phylogeny (Nesse et al., 2016). These are the fast sympathetic–adreno–medullar (SAM) axis, switching mind and body toward immediate action when activated, and the slower hypothalamus–pituitary–adrenal (HPA) axis—which prepares the body and mind for appropriate adaptive action (Porges, 2009; DeVries et al., 2003): fight, flight, freeze or flirt (Mariti et al., 2017). These potential behavioral responses are supported, besides other hormones, by cortisol, which primarily has a metabolic function. Hence, stress modulation strongly and immediately affects metabolic rates, in the same way in humans and their companion animals (Kotrschal, 2013). Via cortisol modulation, blood glucose is either provided for behavioral action, or is stored as insulin or fat for future use. Calming via companion or assistant animals, therefore also strongly affects the metabolism via the down-regulation of cortisol and up-regulation of OT.

Evidently, appropriate stress modulation and management—notably social—is crucially important for the wellbeing, health and longevity of individual humans and other animals. In fact, chronic stimulation of stress systems, particularly HPA, negative affects welfare and health of both the same way. Chronically high cortisol and blood sugar increases the susceptibility to stress-related type II diabetes, cardiovascular problems, brain neuron loss, depression, fear-related mental problems and ultimately, a decreased life span (Knezevic et al., 2023). Hence, social brains generally try to maintain social homoeostasis (above), i.e., favorable social contexts for keeping individual stress modulation within an adaptive range. Whereas social stress may ultimately even be fatal, socio-positive relationships buffer against deleterious stress via emotional support (DeVries et al., 2003). Providing calming and positive motivation is probably at the core of the positive potentials of companion and assistant animals (Beetz et al., 2011; Wheeler and Faulkner, 2015).

Socio-positive interactions between companions—stroking, or even looking into each other’s eyes (Handlin et al., 2011)—activate the so-called “calming system” (Grahn et al., 2021), around OT. Mammalian OT is primarily involved in giving birth, in facilitating maternal bonding to offspring and lactation (Curley and Keverne, 2005). Secondarily, OT supports bonding between romantic partners, as well as in initiating and maintaining the bond between companion animals and their human partners (Beetz et al., 2012; Julius et al., 2012). As OT is a cortisol antagonist, it’s release due to socio-positive interactions mediates calming, with stress-dampening effects persisting for hours to days. Mainly within groups and between social partners, OT supports social interest, trustful relationships and cooperation, buffers against the deleterious effects of stress, and enhances resilience against potential mental problems, including depression and anxiety (DeVries et al., 2003; Grahn et al., 2021). Hence, stress system activation may be counterbalanced by yet another social mechanism, mediating calming via socio-positive interactions between partners, and thereby, creating a safe base for exploring and learning, for cooperating. Essentially, this is a central mechanism of shifting the autonomic nervous system balance from sympathetic toward more parasympathetic (Porges, 2009).

“Social hormones,” such as cortisol or OT, clearly co-vary with relationship contexts in human-human as well as human-animal interactions. However, it should be kept in mind, that species, setting, novelty, sampling decisions, the assays used, general study design, etc., may cause much additional variability, making different studies hard to compare (Rodriguez et al., 2021; Bert et al., 2016; Northrope et al., 2025). Also, the validity of non-invasively measuring OT is still critically discussed (McCullough et al., 2013; Valstad et al., 2017; MacLean et al., 2019; Tabak et al., 2023). Such methodological and analytical caveats should be kept in mind when measuring and interpreting hormones as outcome variables. Of course, hormone assays need to be carefully validated. And for properly interpreting hormone measures, they need to be integrated with behavioral context.

The individual reactivity of the OT system is set early on in human development, within the frame of the attachment/caregiving behavioral system (above). Sensitive and reliable caregiving allows babies to establish their primary social representation in the form of secure attachment (Bowlby, 1979). Early attachment representations channel the systemic and mental reactivity to environmental stimuli and contexts for life. The calming system is a central factor in the mutual emotional social support between companion animals/dogs and their human partners (Grahn et al., 2021; Handlin et al., 2011), and is therefore, key for their wellbeing, welfare, health and also, for trustful and effective pedagogic or therapeutic settings (Kotrschal, 2013, 2014, 2016b).

##### 2.3.1.6 Proper companion animals via domestication and socialization

The shared structures and mechanisms discussed above are essential, but not sufficient to support a social match between humans and their companion animals. It also needs suitable animal partners prepared for companionship and cooperation by domestication and by proper socialization. Virtually all livestock and companion animals are “domesticated,” which means that in contrast to tamed wildlife, they are genetically adapted to live with humans (comp. Purugganan, 2022). Domesticating animals—with dogs being some 30,000 years ahead of other domesticated animals (below)—allowed humans to spread into virtually all habitats, from the equatorial rain forests into the arctic, and to develop a great cultural variety. They are food, partners at war and emotional social supporters. Together with their domesticated animals, humans have radically changed the face of the earth, which is exemplified by the fact that today, more than 95% of the biomass of land-living mammals is made up by humans and their domesticated animals (Bar-On et al., 2018).

The general primary mechanism of domestication is “selection for tameness” (Belyaev, 1972; Wilkins, 2020). This makes animal companions generally more gentle, attentive, workable and less irritable than their wild ancestors. Also, the process of domestication shifts much social interest of these animals from conspecifics toward humans (Mech and Boitani, 2019; Kotrschal, 2022). In fact, it has been recently shown that the social and cooperative orientation of dogs toward humans is wolf heritage (Kotrschal, 2018, 2022; Range and Marshall-Pescini, 2022), but substantially re-oriented toward humans; this was shown by comparing both, free ranging dogs with wolves, and similarly socialized and kept wolves and dogs.

In fact, free-living dog packs are considerably more open to be joined or left, but are less cooperative than wolf packs (Range and Marshall-Pescini, 2022). Under human guidance dogs generally show a greater urge of engaging in complex and diverse cooperation than equally socialized wolves (Range et al., 2019). Consequently, dogs, more than wolves, were emotionally supported by familiar humans, as judged by behavior and heart rate parameters, the calming effect was greater in dogs than in wolves (Jean-Joseph et al., 2020, 2022). In other words, a major factor of wellbeing of both, wolves and dogs, is the quality of the social relationship to their social partners—conspecifics in the case of wolves, but humans more than conspecifics in the case of dogs. But not all of the straightforward predictions of the “selection-for-tameness-hypothesis” necessarily hold. For example, dogs were found to maintain higher, but not lower levels of arousal than comparable wolves (Vasconcellos et al., 2016), even at rest (Jean-Joseph et al., 2020). This may be interpreted as an adaptation of dogs for being permanently at standby to react to the not always predictable human actions.

Such results suggest that the social fit of humans with their domesticated animals—from companion to lifestock—is refined by domestication, probably on both sides, as recently, a kind of human “self-domestication” since the Paleolithic is discussed (Hare et al., 2012). In fact, it seems that ever since humans were selected for becoming nicer and more cooperative—at least within groups—coming with features of the “domestication syndrome” characteristic for other domesticated animals (Wilkins et al., 2014). For example, skulls and skeletons became more delicate and brains arguably smaller in the last 50,000 years (Stibel, 2021). If true, this means that the domestication toward dogs and the potential human self-domestication occurred in parallel; as these two processes were not directly causally linked, they do not qualify as “co-evolution.”

To make trustful and cooperative companions, even domesticated animals need to be properly socialized with humans, preferably in their sensitive ontogenetic periods (Scott and Fuller, 1965; Serpell et al., 2017). In fact, domesticated animals are generally easier socialized, i.e., more sociable with humans, than wildlife. For example, it takes surprisingly little human contact to socialize dog pups (Scott and Fuller, 1965), whereas socializing wolf pups is a major effort (Klinghammer and Goodmann, 1987; Kotrschal, 2018, 2022). It remains speculation, whether human self-domestication (if at all the case) had a similar effect on human sociability with animals. In the light of human biophilia (above), the human universal of living with other animals and a human brain needy of social homoeostasis, it is tempting to assume such a process and effect.

#### 2.3.2 Dogs as an example

Via humans as a vector, the domesticated wolves are among the evolutionarily most successful mammals. An estimated 200,000 wolves remaining in the wild is contrasted by > 1 billion dogs, most of them mainly living in and around large cities; approximately 470 million are kept as companion animals/pets^2^. Hence, dogs, just short of cats, are the most common and widely distributed companion animals, mainly because they share a very similar socio-ecology with humans, including the potential to flexibly and broadly adapt to cultural contexts (Kotrschal, 2016a). Based on the common “social toolbox” (above) similar social mindsets have evolved in parallel in humans and wolves under comparable socio-ecological conditions over the last few million years. Even modern humans and wolves are still similar in lifestyles and social organization, and particularly, in context-dependent flexibility of their social behavior (Gunter et al., 2019; Kotrschal, 2019).

Some 40,000 years ago animistic Eurasian hunter-gatherers came into contact with wolves (Frantz et al., 2016; Kotrschal, 2018; Shipman, 2015). Recent mainstream opinion holds, that in the primary events, wolf pups must have been socialized by alloparental human care (Brumm et al., 2023; Kotrschal, 2022; Mech and Janssens, 2022). In contrast, the “self-domestication hypothesis” suggests that the wolves themselves came to live near humans, mainly because leftovers would be an easy source of food; while this scenario is not *per se* unlikely, it would have resulted in habituated, but not socialized wolves, which would not make cooperative partners and even, by their loss of distance, would have posed a permanent threat to their human hosts.

Based on the surprisingly close social and ecological fit of the two species a culture of living with tame wolves developed in Paleolithic hunter-gatherers (Shipman, 2015). In fact, humans and wolves stayed together, probably soon cooperating over hunting (Kotrschal, 2016b,^2022^; Range and Marshall-Pescini, 2022; Shipman, 2015). Wolf-like dogs (or dog-like wolves) were already around 35,000 years ago, primarily as a result of (implicit) selection for tameness (Belyaev, 1972; Kotrschal, 2022; Wilkins et al., 2014). The similar mindsets of humans and wolves include clan-orientation and complex cooperation in relatively flat hierarchies. During the “Neolithic revolution” (i.e., becoming sedentary) human societies turned more hierarchical. This also affected the selection pressures shaping dog social attitudes. For example, dogs, in contrast to wolves come with an intrinsic disposition to readily and happily submit (Range and Marshall-Pescini, 2022); in contrast to tame wolves dogs therefore expect, and actually require competent human leadership for their wellbeing (Kotrschal, 2016b,^2022^).

Cognitive adaptations of dogs include their decreased abilities to grasp causality, to imitate motor actions of conspecific role models, etc (summarized in Range and Marshall-Pescini, 2022). Their social intelligence seemingly did not decrease (Hare and Woods, 2013; Jardim-Messeder et al., 2017), but was honed to human partners (Heberlein et al., 2016, 2017). Increasing evidence suggests that living in good relationships with companion dogs supports human wellbeing, health and resilience against mental problems (above; Headey et al., 2008; Junça-Silva, 2025; Kotrschal, 2014). Vice versa, dogs benefit in terms of their wellbeing and health when they enjoy cooperative and empathic leadership from side their human partners (Serpell et al., 2017).

In fact, most of the beneficial effects of a good live with companion animals have been found in human-dog research (Mills et al., 2025), probably because dogs are the most common companions for a wide range of activities, are cooperative and easy to handle and investigate, as, for example, compared to cats. They most perfectly fit an active lifestyle, aligning with genuinely human needs. Dog owners usually show more physical activity and enjoy emotional support from their companions, supporting stress buffering and calming, which are important resources for balancing emotionality (Bowlby, 1979; Coan and Maresh, 2014; Kotrschal, 2016a). Dogs act as social lubricants and provide feelings of sense and utility (Bremhorst and Mills, 2021; Kotrschal, 2014). And—as also other close animal companions do—dogs support a diverse microbiome (Salas Garcia et al., 2019). Such, by and large, dog owners are healthier and more resilient to physical and mental problems than socio-economically matched non-dog-owners (Headey et al., 2008; Junça-Silva, 2025; Tóth et al., 2023).

## 3 Some twists of having so much in common

Social companionship between humans and other vertebrates is based on a wealth of shared social mechanisms. In fact, motivation and benefits of living and working with other animals root in human-specific “biophilia” (Fromm, 1981; Wilson, 1984), and the “social homoeostasis” behavior system (Matthews and Tye, 2019). This allows for a close social fit between humans and animals in private keeping and in a variety of pedagogic and therapeutic setting. As humans evolved into socio-cognitive specialists (Kotrschal, 2019), they became particularly needy of an appropriate social environment to make adequate use of their dispositions. Companion animals may be an important components of such a supportive environment (Fine, 2019). Vice versa, companion animals such as dogs seem to have developed “homophilic” mentalities over domestication, being intrinsically interested in humans (Kotrschal, 2022). Hence, the principles of social homoeostasis also seem to be applicable to properly socialized companion animals, as they are intrinsically motivated to seek emotional social support in empathic human partners (DeVries et al., 2003; Grahn et al., 2021; Kotrschal, 2024). Surprisingly similar mindsets have evolved in parallel in humans and wolves, which paved the way for their domestication and for close human-animal partnership.

The substrates for these mental mechanisms allowing for potentially mutually rewarding between-species relationships include the conservative brain social network (Goodson, 2005), the stress systems (Porges, 2009), the oxytocin calming system (Grahn et al., 2021; Handlin et al., 2011), the basic affective systems (Panksepp, 1998, 2005), particularly of attachment and caregiving (Bowlby, 1979; Hinde and Stevenson-Hinde, 1990), as well as the motor and perception systems for affective communication (Tsuboi et al., 2014; Hennessy et al., 1997). In addition to these domains shared on the base of common phylogeny, there are also contributions by parallel evolution: for example, the modern mammalian and bird forebrain centers allowing for complex social behavior by integrating the contributions by other brain areas.

### 3.1 Asymmetric social relationships

Mutually rewarding relationships in the sense of social homoeostasis are possible by activating and utilizing these social mechanisms on both sides. But—as also the case for human-human dyads –relationships are generally not symmetrical, as humans provide the environments dogs and other companion animals are living in and coping with, determined by their attitudes toward animals. This does not mean that the most appropriate way of getting companion animals to cooperate would be “domination” and force. First of all, the common socio-cognitive grounds with them (above) do not justify chauvinistic attitudes of human superiority. Second, domesticated companion animals are intrinsically motivated to cooperate with us, but due to this natural asymmetry the human partners need to provide clear leadership and species-adequate motivation. This includes the occasional setting of limits and empathic consequence in obeying and putting through the rules. Domestication has an important role in making adaptable animal partners. For example, wolf packs in the wild (Mech and Boitani, 2019) show flat hierarchies, with shared decision processes and, with no “Alpha” forcing his/her will through. Consequently, human-socialized wolves cooperate at level with their human partners or even like to take the lead (Range et al., 2019), while dogs prefer to submit and to accept positive leadership (Kotrschal, 2018, 2022; Vasconcellos et al., 2016). Of course, not just the animals adjust to their human partners, such a companionship also significantly feeds back on owner lifestyles, time budgets, social networks, etc (Jean-Joseph et al., 2022; Kotrschal, 2024; Merkouri et al., 2022).

In the interest of the optimal benefits for both sides, in private companionship as well as professional settings, one should be aware that animals/dogs have considerable cognitive potentials (Heberlein et al., 2016, 2017)—they not just “function” on the base of instincts and simple learning. The fact that positive reinforcement training is highly effective in dogs and other animals does not mean that their learning, behavior and decision making is just stimulus-response-based. In alignment with the experience of owners, “do-as-I-do” experiments (Topal et al., 2006) have shown that dogs are capable of mentally representing their environment as a base for their judgments and decision making. The do-as-I-do paradigm in addition to positive reinforcement also adds fun for both sides in dog training, supporting close and trustful bonds.

A way of stimulating anticipatory thinking on the side of the animals is to start with clicker training already early on. Wolf Science Center^3^ trainers, for example, occasionally play a novelty game with their animals, relying a lot um mental representation and thinking. In this game it is up to the animal to offer “something new” in each round, knowing that then, reward will follow a click. For example, a session starts with the “show” command; the animal offers, for example, sitting—click, rewarded. In the second round, the animal again sits down—no click, no reward, because the same action was just offered before—so the wolf or dog circles a tree—click, reward. This game can run for quite some rounds, with the difference to reinforcement training being evident: the animal thinks, i.e., activates the anticipatory resources of their cortical columns creatively offer behavior, showing maximal attentiveness to the human partner.

The cognitive match between humans and dogs even includes passive verbal skills. Particularly gifted dogs can learn up to 1,000 words (Pilley and Hinzmann, 2014). Although no data are available, owner experience suggests, that most companion dogs know the meaning of a few handful of words, the extent of which being probably depends on the kind of verbal communication practiced with a dog Actually, dogs do not need to be actively taught the meaning of words. With particularly “gifted” dogs it was recently shown, that these listen when humans speak with each other and thereby learn the meaning of new words (Dror et al., 2026), just as children would. There is no reason to assume that “normal” companion dogs would tick very differently in this respect. This also underlines that dogs are more or less permanently attentive to humans (Wallis et al., 2014), learn from them, read and interpret our affective expressions (Müller et al., 2015). Actually, dogs judge humans not previously known to them as cooperative or not, as being friendly and approachable, as a potential threat or as being neutral. The dogs’ mentally integrating their environment seem permanently activated (even at rest Jean-Joseph et al., 2020); generally, dogs seem more attentive, particularly toward their social human partners than vice versa.

### 3.2 From anthropomorphising toward empathy and animal welfare concerns

Is anthropomorphizing other animals indeed, highly problematic (Urquiza-Haas and Kotrschal, 2015, 2022)? Or is “anthropomorpho-phobia,” widespread in in science and the humanities, rather a moral prejudice out of social uncertainty toward the “hetero-specific stranger,” based on the lack of knowledge? It depends. First of all, relating to the world, and particularly to other animals, through our own eyes and minds is a hardly avoidable human universal (Brown, 1991). But there is no reason to assume that other animals are too different in this (von Uexküll, 1909). Hence, as much as humans tend to anthropomorphize animals/dogs and recognize them as being friendly or not, happy, focused at work, etc., dogs probably “dogify” humans. And how different are we really? The impressive extent of the shared social toolbox and mental features discussed in this contribution, also indicates, that anthropomorphizing and mentalizing other animals may only be gradually different from what humans permanently do when relating to other humans: interpreting them on the base of their own subjective experiences and attitudes. In the end, anthropomorphizing other animals and being emphatic with them may not be as inappropriate, as generally held, if supplemented by knowledge on their species-specific and individual needs.

So, to a certain extent, human affective empathy—if paired with knowledge—can be trusted in recognizing the needs of animal companions and assistants, in a similar way, as, the affective understanding of these animals toward humans works, because—adequate mutual socialization provided—we are mutually able to read and interpret the affective expressions of hetero-specific social partners. This is a necessary condition for being capable of basic empathy, defined as recognizing the affects and needs of others and responding to them in a complementary way. Such affective and cognitive empathy can be used to support others and/or to manipulate them for one’s own benefit. Humans are certainly capable of this. But it has also been shown that dogs can manipulate human partners on the basis of what they know about their cooperativeness (Heberlein et al., 2016, 2017).

Empathy-based knowledge is particularly important not to overburden companion and assistant animals in the interest of human benefits. In alignment with IAHAIO declarations^1^, these animals should symmetrically profit from collaborating with their human partners and must not be exploited. Particularly in the notoriously compliant dogs, subtle signs of discomfort and stress are easily missed. Stress behavior monitoring in the interest of animal welfare seems particularly important in institutional animal-assisted contexts, which also require particular care in comparing outcomes across programs and studies (Fine et al., 2019; Glenk, 2017; Glenk and Foltin, 2021; Meers et al., 2022).

Empathy in combination with knowledge of the specific needs of companions/partners allows for rewarding relationships between humans and dogs—even from the dog’s side, as the animal learns to judge their human partner and to react appropriately in order to optimize the benefits from and pleasantness of a particular partnership (in the sense of social homoeostasis; Matthews and Tye, 2019). But the human-dog/animal partnership may be profoundly asymmetric in this respect, not the least because humans are more burdened by social and moral uncertainty about the proper handling of their dogs than vice versa. Humans are cognitive ruminants, which may inhibit social instincts and clear leadership. Dogs, in contrast, are pretty straightforward in utilizing such uncertainty of their human partner to their own benefit. By nature, they are consequent at conditioning their human partners. In principle, social bargaining and manipulating the partner in ones’ own interest is an integrative feature of all social partnerships (Sluzki, 2010), be it between humans and also, between humans and companion animals/dogs. But this remains within limits in functional, positively rewarding partnerships. For example, if dogs are forced to take over at least partial control by disorganized attachment/interactions and dysfunctional human leadership (Solomon et al., 2019), this is neither in the interest of dog nor human welfare and safety.

### 3.3 Dark sides

No benefits without costs: The dark sides of the human–dog collusion include that occasionally, humans, particularly children, are injured or even killed by dogs (Kotrschal, 2014), often as a result of interspecies social conflicts and misunderstandings. Also, particularly at poor or lacking veterinary care dogs can be zoonotic vectors, transmitting rabies, leishmaniosis, leptospirosis, etc. Street dogs, in particular, may be a public health issue. Based on a WHO estimate, dogs may cause well more than 30,000 casualties per year globally^4^, mainly as a major vector of rabies, by causing accidents, etc. From a dog’s perspective, a failed relationship may get them on the street, in a shelter or even death, for example by euthanasia.

## 4 Conclusion

Mainstream occidental philosophy has generally over-emphasized the socio-cognitive gap between humans and other animals. In line with the “Darwinian continuum,” recent scientific results indicate, that dogs and other companion or assistant animals are socially not fundamentally different from humans, due to a “shared social toolbox” and due to mentally adapting to humans by domestication. This also supports recent results indicating that the social and cooperative orientation of dogs toward humans is basically wolf heritage (Range and Virányi, 2015), but was re-orientated toward humans by domestication. Wolves/dogs, and, above all, humans, have evolved into complex socio-cognitive specialists. Therefore, living in social homoeostasis, i.e., in adequate interspecies social relationships is a core component and trigger of the kind of social physiology supporting welfare and even health, of the companion animals, as well as of their human partners—but it has to be kept in mind, that the general scientific evidence for this still remains ambiguous. This is also key for the effectiveness of animal-assisted pedagogic or therapeutic settings.

## Footnotes

1
https://iahaio.org/best-practice/declarations/

2 World Animal Foundation: https://worldanimalfoundation.org/dogs/

3
https://www.wolfscience.at/

4
www.statista.com/statistics/448169/deadliest-creatures-in-the-world-by-number-of-human-deaths/

## References

[B1] Adkins-Regan E. (2009). Neuroendocrinology of social behavior. *ILAR J.* 50 5–14. 10.1093/ilar.50.1.5 19106448

[B2] Adolphs R. (2003). Cognitive neuroscience of human social behaviour. *Nat. Rev. Neurosci.* 4 165–178. 10.1038/nrn1056 12612630

[B3] Albuquerque N. Resende B. (2023). Dogs functionally respond to and use emotional information from human expressions. *Evol. Hum. Sci.* 5:e2. 10.1017/ehs.2022.57 37587944 PMC10426098

[B4] Allen T. A. Fortin N. J. (2013). The evolution of episodic memory. *Proc. Natl. Acad. Sci. U. S. A.* 110 10379–10386. 10.1073/pnas.1301199110 23754432 PMC3690604

[B5] Allsop A. S. Tye K. M. Krystal J. H. (2025). Social homeostasis: A new paradigm for mental health diagnosis and treatment. *Biol. Psychiatry* 97 932–935. 10.1016/j.biopsych.2025.03.007 40316377

[B6] Amodio D. M. (2019). Social cognition 2.0: An interactive memory systems account. *Trends Cogn. Sci.* 23 21–33. 10.1016/j.tics.2018.10.002 30466793

[B7] Applebaum J. W. Shieu M. M. McDonald S. E. Dunietz G. L. Braley T. J. (2023). The impact of sustained ownership of a pet on cognitive health: A population-based study. *J. Aging Health* 35 230–241. 10.1177/08982643221122641 36006805 PMC10280126

[B8] Barcelos A. M. Kargas N. Maltby J. Mills D. S. (2023). Potential psychosocial explanations for the impact of pet ownership on human well-being: Evaluating and expanding current hypotheses. *Hum. Anim. Interact.* 2023:8. 10.1079/hai.2023.0008

[B9] Bar-On Y. M. Phililips R. Milo R. (2018). The biomass distribution on earth. *Proc. Natl. Acad. Sci. U. S. A.* 115 6506–6511. 10.1073/pnas.1711842115 29784790 PMC6016768

[B10] Bauman A. Owen K. B. Torske M. O. Ding D. Krokstad S. Stamatakis E. (2020). Does dog ownership really prolong survival? A revised meta-analysis and reappraisal of the evidence. *Circ. Cardiovasc. Qual. Outcomes* 13:e006907. 10.1161/CIRCOUTCOMES.120.006907 33079585

[B11] Beetz A. M. (2017). Theories and possible processes of action in animal assisted interventions. *Appl. Dev. Sci.* 21 139–149. 10.1080/10888691.2016.1262263

[B12] Beetz A. M. Kotrschal K. Turner D. C. Hediger K. Uvnäs-Moberg K. Julius H. (2011). The effect of a real dog, toy dog and friendly person on insecurely attached children during a stressful task: An exploratory study. *Anthrozoös* 24 349–368. 10.2752/175303711X13159027359746

[B13] Beetz A. M. Uvnäs-Moberg K. Julius H. Kotrschal K. (2012). Psychosocial and psychophysiological effects of human-animal interactions: The possible role of oxytocin. *Front. Psychol.* 3:234. 10.3389/fpsyg.2012.00234 22866043 PMC3408111

[B14] Belyaev D. K. (1972). The Wilhelmine E. Key 1978 invitational lecture. Destabilizing selection as a factor in domestication. *Heredity* 70 301–308. 10.1093/oxfordjournals.jhered.a109263 528781

[B15] Bennett M. S. (2021). What behavioral abilities emerged at key milestones in human brain evolution? 13 hypotheses on the 600-million-year phylogenetic history of human intelligence. *Front. Psychol.* 12:685853. 10.3389/fpsyg.2021.685853 34393912 PMC8358274

[B16] Bennett M. S. (2023). *A Brief History of Intelligence.* New York, NY: Harper Collins.

[B17] Bernard L. C. Mills M. Swenson L. Walsh R. P. (2005). An evolutionary theory of human motivation. *Genet. Soc. Gen. Psychol. Monogr.* 131 129–184. 10.3200/MONO.131.2.129-184 16779946

[B18] Berns G. (2013). *How Dogs Love Us: A Neuroscientist and His Adopted Dog Decode the Canine Brain.* Boston, MA: Hughton Mifflin Harcourt.

[B19] Bert F. Gualano M. R. Camussi E. Pieve G. Voglino G. Siliquini R. (2016). Animal assisted intervention: A systematic review of benefits and risks. *Eur. J. Integr. Med.* 8 695–706. 10.1016/j.eujim.2016.05.005 32362955 PMC7185850

[B20] Bowlby J. (1979). The Bowlby-Ainsworth attachment theory. *Behav. Brain Sci.* 2 637–638. 10.1017/S0140525X00064955

[B21] Bowlby J. (1969/1982). *Attachment and Loss Vol. 1: Attachment.* New York, NY: Basic Books.

[B22] Brandt L. Liu S. Heim C. Heinz A. (2022). The effects of social isolation stress and discrimination on mental health. *Transl. Psychiatry* 12:398. 10.1038/s41398-022-02178-4 36130935 PMC9490697

[B23] Bremhorst A. Mills D. (2021). “Working with companion animals, and especially dogs, in therapeutic and other AAI settings,” in *The Welfare of Animals in Animal-Assisted Interventions: Foundations and Best Practice Methods*, eds Peralta J. M. Fine A. H. (Cham: Springer International Publishing), 191–217.

[B24] Brown D. E. (1991). *Human Universals.* New York, NY: McGraw-Hill.

[B25] Brumm A. Germonpré M. Koungoulos L. (2023). The human-initiated model of wolf domestication–an expansion based on human-dingo relations in Aboriginal Australia. *Front. Psychol.* 14:1082338. 10.3389/fpsyg.2023.1082338 37205085 PMC10187142

[B26] Capela e Silva F. Kieson E. Stergiou A. N. Pereira-Figueiredo I. (2024). Editorial: How animals affect us: Examining the influence of human-animal interactions on human’s health. *Front. Vet. Sci.* 11:1509960. 10.3389/fvets.2024.1509960 39582885 PMC11581935

[B27] Christakis N. A. (2019). *Blueprint. The Evolutionary Origins of a Good Society.* New York, NY: Little, Brown Sparks.

[B28] Cimarelli G. Marshall-Pescini S. Range F. Berghaenel A. Viranyi Z. (2021). Relationship quality affects social stress buffering in dogs and wolves. *Anim. Behav.* 178 127–140. 10.1016/j.anbehav.2021.06.008

[B29] Cimarelli G. Range F. Hann K. Kotrschal K. Gácsi M. Virányi Z. (2024). Both humans and conspecifics provide social support to dog and wolf puppies. *Anim. Behav.* 209 129–141. 10.1016/j.anbehav.2024.01.001

[B30] Clayton N. S. Griffiths D. P. Emery N. J. Dickinson A. (2001). Elements of episodic-like memory in animals. *Philos. Trans. R. Soc. Lond. B Biol. Sci.* 356 1483–1491. 10.1098/rstb.2001.0947 11571038 PMC1088530

[B31] Coan J. A. Maresh E. L. (2014). “Social baseline theory and the social regulation of emotion,” in *Handbook of Emotion Regulation*, ed. Gross J. J. (New York, NY: The Guilford Press), 221–236.

[B32] Curley J. P. Keverne E. B. (2005). Genes, brains and mammalian social bonds. *Trends Ecol. Evol.* 20 561–567. 10.1016/j.tree.2005.05.018 16701435

[B33] Damasio A. R. Damasio H. (eds). (2012). *Neurobiology of Decision-Making.* Berlin: Springer Science & Business Media.

[B34] Darwin C. (1897). *The Expressions of the Emotions in Man and Animals.* New York, NY: D. Appleton and Company.

[B35] De Waal F. C. (1982). *Chimpanzee Politics: Power and Sex Among Apes.* Baltimore: John Hopkins University Press.

[B36] DeLaurier A. (2019). Evolution and development of the fish jaw skeleton. *Wiley Interdiscip. Rev. Dev. Biol.* 8:e337. 10.1002/wdev.337 30378758 PMC8299565

[B37] DeLoache J. S. Pickard M. B. LoBue V. (2011). “How very young children think about animals,” in *How Animals Affect Us: Examining the Influences of Human-Animal Interaction on Child Development and Human Health*, eds McCune S. Griffin S. J. A. Maholmes V. (Washington, DC: American Psychological Association), 85–99.

[B38] DeVries A. C. Glasper E. R. Detillion C. E. (2003). Social modulation of stress responses. *Physiol. Behav.* 79 399–407. 10.1016/s0031-9384(03)00152-5 12954434

[B39] Diamond A. (2013). Executive functions. *Ann. Rev. Psychol.* 64 135–168. 10.1146/annurev-psych-113011-143750 23020641 PMC4084861

[B40] Dror S. Miklósi Á. Morvai B. Nãstase A. S. Fugazza C. (2026). Dogs with a large vocabulary of object labels learn new labels by overhearing like 1.5-year-old infants. *Science* 391 160–163. 10.1126/science.adq5474 41505543

[B41] Eibl-Eibesfeldt I. (1967). *Grundriss der Vergleichenden Verhaltensforschung.* München: Piper.

[B42] Ekman P. (1999). “Basic emotions,” in *Handbook of Cognition and Emotion*, Vol. 98 eds Dalgleish T. Power M. (Sussex: John Wiley & Sons), 45–60.

[B43] Fine A. H. Beck A. M. Ng Z. (2019). The state of animal-assisted interventions: Addressing the contemporary issues that will shape the future. *Int. J. Environ. Res. Public Health* 16:3997. 10.3390/ijerph16203997 31635430 PMC6843928

[B44] Fine A. H. (ed.). (2019). *Handbook on Animal-Assisted Therapy: Foundations and Guidelines for Animal-Assisted Interventions.* Amsterdam: Academic Press.

[B45] Fine A. H. Mueller M. K. Ng Z. Y. Griffin T. C. Tedeschi P. (2025). “Final thoughts: The editors’ reflections on the state of animal-assisted interventions,” in *Handbook on Animal-Assisted Therapy*, ed. Fine A. H. (Amsterdam: Academic Press), 489–496.

[B46] Fine A. H. Weaver S. J. (2018). “The human-animal bond and animal-assisted intervention,” in *Oxford Textbook of Nature and Public Health: The Role of Nature in Improving the Health of a Population*, eds van den Bosch M. Bird W. (Oxford: Oxford University Press), 132–138.

[B47] Frantz L. A. F. Mullin V. E. Pionnier-Capitan M. Lebrasseur O. Ollivier M. Perri A. et al. (2016). Genomic and archaeological evidence suggest a dual origin of domestic dogs. *Science* 352 1228–1231. 10.1126/science.aaf3161 27257259

[B48] Friedmann E. Barker S. B. Allen K. M. (2011). “Physiological correlates of health benefits from pets,” in *How Animals Affect us: Examining the Influences of Human–Animal Interaction on Child Development and Human Health*, eds McCardle P. McCune S. Griffin J. A. Maholmes V. (Washington, DC: American Psychological Association), 163–182. 10.1037/12301-009

[B49] Frigon A. (2012). Central pattern generators of the mammalian spinal cord. *Neuroscientist* 18 56–69. 10.1177/1073858410396101 21518815

[B50] Fromm E. (1981). *Die Seele des Menschen. Ihre Fähigkeit zum Guten und zum Bösen.* München: Ullstein.

[B51] Gácsi M. Gyõri B. Miklósi Á. Virányi Z. Kubinyi E. Topál J. et al. (2005). Species-specific differences and similarities in the behavior of hand-raised dog and wolf pups in social situations with humans. *Dev. Psychobiol.* 47 111–122. 10.1002/dev.20082 16136572

[B52] Gilbey A. Tani K. (2015). Companion animals and loneliness: A systematic review of quantitative studies. *Anthrozoös* 28 181–197. 10.2752/089279315X14219211661615

[B53] Glenk L. M. (2017). Current perspectives on therapy dog welfare in animal-assisted interventions. *Animals* 7:7. 10.3390/ani7020007 28157145 PMC5332928

[B54] Glenk L. M. Foltin S. (2021). Therapy dog welfare revisited: A review of the literature. *Vet. Sci.* 8:226. 10.3390/vetsci8100226 34679056 PMC8538106

[B55] Goodson J. L. (2005). The vertebrate social behavior network: Evolutionary themes and variations. *Horm. Behav.* 48 11–22. 10.1016/j.yhbeh.2005.02.003 15885690 PMC2570781

[B56] Grahn P. Ottosson J. Uvnäs-Moberg K. (2021). The oxytocinergic system as a mediator of anti-stress and instorative effects induced by nature: The calm and connection theory. *Front. Psychol.* 12:617814. 10.3389/fpsyg.2021.617814 34290636 PMC8286993

[B57] Gunter L. M. Feuerbacher E. N. Gilchrist R. J. Wynne C. D. (2019). Evaluating the effects of a temporary fostering program on shelter dog welfare. *PeerJ* 7:e6620. 10.7717/peerj.6620 30944778 PMC6441318

[B58] Güntürkün O. (2005). The avian ‘prefrontal cortex’ and cognition. *Curr. Opin. Neurobiol.* 15 686–693. 10.1016/j.conb.2005.10.003 16263260

[B59] Handlin L. Hydbring-Sandberg E. Nilsson A. Ejdebäck M. Jansson A. Uvnäs-Moberg K. (2011). Short-term interaction between dogs and their owners: Effects on oxytocin, cortisol, insulin and heart rate—An exploratory study. *Anthrozoös* 24 301–316. 10.2752/175303711X13045914865385

[B60] Hansen P. R. König H. H. Hajek A. (2024). Pet ownership and psychosocial factors in adults aged 40 years and over: Results of a large nationally representative longitudinal survey. *Societies* 14:132. 10.3390/soc14080132

[B61] Hardie S. Mai D. L. Howell T. J. (2023). Social support and wellbeing in cat and dog owners, and the moderating influence of pet–owner relationship quality. *Anthrozoös* 36 891–907. 10.1080/08927936.2023.2182029

[B62] Hare B. Wobber V. Wrangham R. (2012). The self-domestication hypothesis: Evolution of bonobo psychology is due to selection against aggression. *Anim. Behav.* 83 573–585. 10.1016/j.anbehav.2011.12.007

[B63] Hare B. Woods V. (2013). *The Genius of Dogs: Discovering the Unique Intelligence of Man’s Best Friend.* New York, NY: Simon and Schuster.

[B64] Headey B. Na F. Zheng R. (2008). Pet dogs benefit owners’ health: A ‘natural experiment’ in China. *Soc. Indic. Res.* 87 481–493. 10.1007/s11205-007-9142-2

[B65] Heberlein M. T. E. Manser M. B. Turner D. C. (2017). Deceptive-like behaviour in dogs (*Canis familiaris*). *Anim. Cogn.* 20 511–520. 10.1007/s10071-017-1078-6 28251387

[B66] Heberlein M. T. E. Turner D. C. Range F. Viranyi Z. (2016). The absence of reward induces inequity aversion in dogs. *Anim. Behav.* 122 59–66. 10.1016/j.anbehav.2016.09.023 27974861 PMC5140004

[B67] Hennessy M. B. Davis H. N. Williams M. T. Mellott C. Douglas C. W. (1997). Plasma cortisol levels of dogs at a county animal shelter. *Physiol. Behav.* 62 485–490. 10.1016/s0031-9384(97)80328-9 9272654

[B68] Herculano-Houzel S. (2016). *The Human Advantage: A New Understanding of how our Brain Became Remarkable.* Cambridge, MA: MIT Press.

[B69] Herzog H. (2011). The impact of pets on human health and psychological well-being: Fact, fiction, or hypothesis? *Curr. Dir. Psychol. Sci.* 20 236–239. 10.1177/0963721411415220

[B70] Hinde R. A. Stevenson-Hinde J. (1990). Attachment: Biological, cultural and individual desiderata. *Hum. Dev.* 33 62–72. 10.1159/000276503

[B71] Hinde R. A. Stevenson-Hinde J. (2006). “Perspectives on attachment,” in *Attachment Across the Life Cycle*, eds Stevenson-Hinde J. Marris P. (London: Routledge), 60–73.

[B72] Hoagwood K. E. Acri M. Morrissey M. Peth-Pierce R. (2017). Animal-assisted therapies for youth with or at risk for mental health problems: A systematic review. *Appl. Dev. Sci.* 21 1–13. 10.1080/10888691.2015.1134267 28798541 PMC5546745

[B73] Jablonka E. Lamb M. (2014). *Evolution in Four Dimensions.* Cambridge, MA: MIT Press.

[B74] Jardim-Messeder D. Lambert K. Noctor S. Pestana F. M. de Castro Leal M. E. Bertelsen M. F. et al. (2017). Dogs have the most neurons, though not the largest brain: Trade-off between body mass and number of neurons in the cerebral cortex of large carnivoran species. *Front. Neuroanat.* 11:118. 10.3389/fnana.2017.00118 29311850 PMC5733047

[B75] Jean-Joseph H. Dooey G. Kotrschal K. (2022). Diurnal activity patterns of equally socialized and kept wolves, *Canis lupus*, and dogs, *Canis lupus familiaris*. *Anim. Behav.* 190 41–52. 10.1016/j.anbehav.2022.05.009

[B76] Jean-Joseph H. Kortekaas K. Range F. Kotrschal K. (2020). Context-specific arousal during resting in wolves and dogs: Effects of domestication? *Front. Psychol.* 11:568199. 10.3389/fpsyg.2020.568199 33329204 PMC7732590

[B77] Julius H. Beetz A. Kotrschal K. Turner D. Uvnäs-Moberg K. (2012). *Attachment to Pets. An Integrative View of Human-Animal Relationships with Implications for Therapeutic Practice.* Göttingen: Hogrefe.

[B78] Junça-Silva A. (2025). Beyond companionship: Pets are non-human beings that protect humans’ health. *Curr. Psychol.* 44 3627–3642. 10.1007/s12144-025-07354-5

[B79] Kanat-Maymon Y. Antebi A. Zilcha-Mano S. (2016). Basic psychological need fulfillment in human–pet relationships and well-being. *Pers. Individ. Dif.* 92 69–73. 10.1016/j.paid.2015.12.025

[B80] Kellert S. R. Wilson E. O. (1993). *The Biophilia Hypothesis.* Washington DC: Islands Press.

[B81] Klinghammer E. Goodmann P. A. (1987). “Socialization and management of wolves in captivity,” in *Man and Wolf: Advances, Issues, and Problems in Captive Wolf Research*, ed. Frank H. (Dordrecht: Dr. W. Junk Publishers), 31–59.

[B82] Knezevic E. Nenic K. Milanovic V. Knezevic N. N. (2023). The role of cortisol in chronic stress, neurodegenerative diseases, and psychological disorders. *Cells* 12:2726. 10.3390/cells12232726 38067154 PMC10706127

[B83] Kotrschal K. (2013). “Emotions are at the core of individual social performance,” in *Emotions of Animals and Humans: Comparative Perspectives. The Science of the Mind*, eds Watanabe S. Kucvzaj S. K. (Tokyo: Springer), 3–21.

[B84] Kotrschal K. (2014). *Einfach Beste Freunde. Warum Menschen und Andere Tiere Einander Verstehen.* Vienna: Brandstätter.

[B85] Kotrschal K. (2016a). “Do companion animals support social, emotional and cognitive development of children?,” in *The Social Neuroscience of Human-Animal Interaction*, eds Mc Cune S. Esposito L. Gee N. R. McCardle P. (Washington, DC: American Psychological Association), 73–86.

[B86] Kotrschal K. (2016b). *Hund und Mensch.* Vienna: Brandstätter.

[B87] Kotrschal K. (2018). How wolves turned into dogs and how dogs are valuable in meeting human social needs. *People Anim. Int. J. Res. Pract.* 1 1–18.

[B88] Kotrschal K. (2019). *Mensch. Woher wir Kommen, wer wir Sind, Wohin wir Gehen.* Vienna: Brandstätter.

[B89] Kotrschal K. (2022). *Der Wolf und wir: Wie aus ihm Unser Erstes Haustier Wurde—und Warum Seine Rückkehr Chancen Bietet.* Vienna: Brandstätter.

[B90] Kotrschal K. (2023). Wolf–dog–human: Companionship based on common social tools. *Animals* 13:2729. 10.3390/ani13172729 37684993 PMC10486892

[B91] Kotrschal K. (2024). *Warum Hunde uns zu Besseren Menschen Machen.* Vienna: Brandstätter.

[B92] Kotrschal K. Ortbauer B. (2003). Behavioral effects of the presence of a dog in a classroom. *Anthrozoös* 16 147–159. 10.2752/089279303786992170

[B93] Kotrschal K. Scheiber I. B. R. Hirschenhauser K. (2023). “Individual performance in complex social systems: The greylag goose example,” in *Animal Behaviour: An Evolutionary Perspective*, ed. Kappeler P. (Heidelberg: Springer), 121–148.

[B94] Kovács K. E. Balogh É. Z. Lovas B. Boris P. Nagy B. E. (2024). The role of animal-assisted programs in physical health improvement of children and adolescents with special education needs-a systematic review. *BMC Public Health* 24:824. 10.1186/s12889-024-18326-y 38491498 PMC10943833

[B95] Kramer C. K. Mehmood S. Suen R. S. (2019). Dog ownership and survival: A systematic review and meta-analysis. *Circ. Cardiovasc. Qual. Outcomes* 12:e005554. 10.1161/CIRCOUTCOMES.119.005554 31592726

[B96] Kretzler B. König H. H. Hajek A. (2022). Pet ownership, loneliness, and social isolation: A systematic review. *Soc. Psychiatry Psychiatr. Epidemiol.* 57 1935–1957. 10.1007/s00127-022-02332-9 35816194 PMC9272860

[B97] Lesch R. Kotrschal K. Kitchener A. C. Fitch W. T. Kotrschal A. (2022). The expensive-tissue hypothesis may help explain brain-size reduction during domestication. *Commun. Integr. Biol.* 15 190–192. 10.1080/19420889.2022.2101196 35957842 PMC9359384

[B98] Levita L. Bois C. Healey A. Smyllie E. Papakonstantinou E. Hartley T. et al. (2014). The Behavioural Inhibition System, anxiety and hippocampal volume in a non-clinical population. *Biol. Mood Anxiety Disord.* 4:4. 10.1186/2045-5380-4-4 24607258 PMC4007806

[B99] Liu S. (2024). “The basic trajectory of theoretical evolution: Balancing the individual and the community,” in *The Contemporary Evolution and Reform of Utilitarianism*, ed. S. Liu (Singapore: Springer Nature), 127–174. 10.1007/978-981-99-7363-7_5

[B100] Lorenz K. (1978). *Vergleichende Verhaltensforschung: Grundlagen der Ethologie.* New York, NY: Springer.

[B101] Louv R. (2005). *Last Child in the Woods: Saving Our Children from Nature-Deficit Disorder.* New York, NY: Alonquin Books.

[B102] MacLean E. L. Wilson S. R. Martin W. L. Davis J. M. Nazarloo H. P. Carter C. S. (2019). Challenges for measuring oxytocin: The blind men and the elephant? *Psychoneuroendocrinology* 107 225–231. 10.1016/j.psyneuen.2019.05.018 31163380 PMC6634994

[B103] Malinowski K. Yee C. Tevlin J. M. Birks E. K. Durando M. M. Pournajafi-Nazarloo H. et al. (2018). The effects of equine assisted therapy on plasma cortisol and oxytocin concentrations and heart rate variability in horses and measures of symptoms of post-traumatic stress disorder in veterans. *J. Equine Vet. Sci.* 64 17–26. 10.1016/j.jevs.2018.01.011 30973147

[B104] Mariti C. Pierantoni L. Sighieri C. Gazzano A. (2017). Guardians’ perceptions of dogs’ Welfare and behaviors related to visiting the veterinary clinic. *J. Appl. Anim. Welf. Sci.* 20 24–33. 10.1080/10888705.2016.1216432 27712096

[B105] Marler P. (2014). “The instinct to learn,” in *The Epigenesis of Mind*, eds Carey S. Gelman R. (London: Tayler & Francis), 37–66.

[B106] Marsha N. Scheelea D. Feinstein J. S. Gerhardtd H. Strange S. Maier W. et al. (2017). Oxytocin-enforced norm compliance reduces xenophobic outgroup rejection. *Proc. Natl. Acad. Sci. U. S. A.* 114 9314–9319. 10.1073/pnas.1705853114 28808030 PMC5584433

[B107] Matthews G. A. Tye K. M. (2019). Neural mechanisms of social homeostasis. *Ann. N. Y. Acad. Sci.* 1457 5–25. 10.1111/nyas.14016 30875095 PMC7593988

[B108] McCullough M. E. Churchland P. S. Mendez A. J. (2013). Problems with measuring peripheral oxytocin: Can the data on oxytocin and human behavior be trusted? *Neurosci. Biobehav. Rev.* 3 1485–1492. 10.1016/j.neubiorev.2013.04.018 23665533

[B109] McGetrick J. Range F. (2018). Inequity aversion in dogs: A review. *Learn. Behav.* 46 479–500. 10.3758/s13420-018-0338-x 30105647 PMC6268111

[B110] McNicholas J. Gilbey A. Rennie A. Ahmedzai S. Dono J. A. Ormerod E. (2005). Pet ownership and human health: A brief review of evidence and issues. *BMJ* 331 1252–1254. 10.1136/bmj.331.7527.1252 16308387 PMC1289326

[B111] Mech L. D. Boitani L. (eds). (2019). *Wolves: Behavior, Ecology, and Conservation.* Chicago, IL: University of Chicago Press.

[B112] Mech L. D. Janssens L. A. (2022). An assessment of current wolf *Canis lupus* domestication hypotheses based on wolf ecology and behaviour. *Mamm. Rev.* 52 304–314. 10.1111/mam.12273

[B113] Meers L. L. Contalbrigo L. Samuels W. E. Duarte-Gan C. Berckmans D. Laufer S. J. et al. (2022). Canine-assisted interventions and the relevance of welfare assessments for human health, and transmission of zoonosis: A literature review. *Front. Vet. Sci.* 9:899889. 10.3389/fvets.2022.899889 35782560 PMC9247644

[B114] Meixner J. Kotrschal K. (2022). Animal-assisted interventions with dogs in special education—A systematic review. *Front. Psychol.* 13:876290. 10.3389/fpsyg.2022.876290 35712211 PMC9197485

[B115] Melson G. F. (2003). Child development and the human-companion animal bond. *Am. Behav. Sci.* 47 31–39. 10.1177/0002764203255210

[B116] Melson G. F. Fine A. H. VonLintel J. F. (2025). “Animals in the lives of children,” in *Handbook on Animal-Assisted Therapy*, ed. Fine A. (Cambridge, MA: Academic Press), 301–322.

[B117] Merkouri A. Graham T. M. O’Haire M. E. Purewal R. Westgarth C. (2022). Dogs and the good life: A cross-sectional study of the association between the dog–owner relationship and owner mental wellbeing. *Front. Psychol.* 13:903647. 10.3389/fpsyg.2022.903647 35923726 PMC9341998

[B118] Mills D. S. Rogers J. Hall S. Kerulo G. Bremhorst A. (2025). “Getting the right dog for the right job for animal-assisted activities, interventions, and therapies: Essential understanding of dog behavior and ethology for those working with dogs to help others,” in *Handbook on Animal-Assisted Therapy*, ed. Fine A. (Cambridge, MA: Academic Press), 183–202.

[B119] Miyake A. Friedman N. P. (2012). The nature and organization of individual differences in executive functions: Four general conclusions. *Curr. Dir. Psychol. Sci.* 21 8–14. 10.1177/0963721411429458 22773897 PMC3388901

[B120] Moshfeghinia R. Rostamipourfard S. Kamran M. Mostafavi S. Golabi F. Ayano G. et al. (2025). Pet ownership and risk of depression: A systematic review and meta-analysis. *Ann. Gen. Psychiatry* 24:66. 10.1186/s12991-025-00600-x 41194144 PMC12590595

[B121] Müller C. A. Schmitt K. Barber A. L. Huber L. (2015). Dogs can discriminate emotional expressions of human faces. *Curr. Biol.* 25 601–605. 10.1016/j.cub.2014.12.055 25683806

[B122] Nesse R. M. Bhatnagar S. Ellis B. (2016). “Evolutionary origins and functions of the stress response system,” in *Stress: Concepts, Cognition, Emotion, and Behaviour*, ed. Fink G. (Cambridge, MA: Academic Press), 95–101.

[B123] Northrope K. Shnookal J. Ruby M. B. Howell T. J. (2025). The relationship between attachment to pets and mental health and wellbeing: A systematic review. *Animals* 15:1143. 10.3390/ani15081143 40281976 PMC12023967

[B124] Oberliessen L. Kalenscher T. (2019). Social and non-social mechanisms of inequity aversion in non-human animals. *Front. Behav. Neurosci.* 13:133. 10.3389/fnbeh.2019.00133 31293399 PMC6598742

[B125] O’Connell L. A. Hofmann H. A. (2011). The vertebrate mesolimbic reward system and social behavior network: A comparative synthesis. *Comp. Neurol.* 519 3599–3639. 10.1002/cne.22735 21800319

[B126] O’Haire M. (2010). Companion animals and human health: Benefits, challenges, and the road ahead. *J. Vet. Behav.* 5 226–234. 10.1016/j.jveb.2010.02.002

[B127] Panksepp J. (1998). *Affective Neuroscience.* New York, NY: Oxford University Press.

[B128] Panksepp J. (2005). Affective consciousness: Core emotional feelings in animals and humans. *Conscious. Cogn.* 14 30–80. 10.1016/j.concog.2004.10.004 15766890

[B129] Parsons C. E. Landberger C. Purves K. L. Young K. S. (2024). No beneficial associations between living with a pet and mental health outcomes during the COVID-19 pandemic in a large UK longitudinal sample. *Ment. Health Prev.* 35:200354. 10.1016/j.mhp.2024.200354

[B130] Pietromonaco P. R. Powers S. I. (2015). Attachment and health-related physiological stress processes. *Curr. Opin. Psychol.* 1 34–39. 10.1016/j.copsyc.2014.12.001 25729755 PMC4341899

[B131] Pilley J. W. Hinzmann H. (2014). *Chaser: Unlocking the Genius of the Dog who Knows 1000 Words.* New York, NY: Simon and Schuster.

[B132] Pitt S. J. Gunn A. (2024). The one health concept. *Br. J. Biomed. Sci.* 81:12366. 10.3389/bjbs.2024.12366 38434675 PMC10902059

[B133] Porges S. W. (2009). The polyvagal theory: New insights into adaptive reactions of the autonomic nervous system. *Cleve. Clin. J. Med.* 76 86–90. 10.3949/ccjm.76.s2.17 19376991 PMC3108032

[B134] Purugganan M. D. (2022). What is domestication? *Trends Ecol. Evol.* 37 663–671. 10.1016/j.tree.2022.04.006 35534288

[B135] Range F. Horn L. Viranyi Z. Huber L. (2009). The absence of reward induces inequity aversion in dogs. *Proc. Natl. Acad. Sci. U. S. A.* 106 340–345. 10.1073/pnas.0810957105 19064923 PMC2629244

[B136] Range F. Marshall-Pescini S. (2022). *Wolves and Dogs. Between Myth and Science.* Berlin: Springer.

[B137] Range F. Marshall-Pescini S. Kratz C. Virányi Z. (2019). Wolves lead and dogs follow, but they both cooperate with humans. *Sci. Rep.* 9:3796. 10.1038/s41598-019-40468-y 30846770 PMC6405935

[B138] Range F. Virányi Z. (2015). Tracking the evolutionary origins of dog-human cooperation: The “Canine Cooperation Hypothesis”. *Front. Psychol.* 5:1582. 10.3389/fpsyg.2014.01582 25642203 PMC4295532

[B139] Reilly O. T. Somerville L. H. Hecht E. E. (2024). Mechanisms of social attachment between children and pet dogs. *Animals* 14:3036. 10.3390/ani14203036 39457966 PMC11505475

[B140] Rodriguez K. E. Herzog H. Gee N. R. (2021). Variability in human-animal interaction research. *Front. Vet. Sci.* 7:619600. 10.3389/fvets.2020.619600 33521092 PMC7843787

[B141] Russell B. (1950). *Unpopular Essays.* New York, NY: Simon & Schuster.

[B142] Salas Garcia M. C. Schorr A. R. Arnold W. Fei N. Gilbert J. A. (2019). “Pets as a novel microbiome-based therapy,” in *Pets as Sentinels, Forecasters and Promoters of Human Health* (Cham: Springer International Publishing), 245–267.

[B143] Schleidt W. (1974). How “fixed” is the fixed action pattern? *Z. Tierpsychol.* 36 184–211. 10.1111/j.1439-0310.1974.tb02131.x 4467663

[B144] Schöberl I. Wedl M. Bauer B. Day J. Möstl E. Kotrschal K. (2012). Effects of owner–dog relationship and owner personality on cortisol modulation in human–dog dyads. *Anthrozoös* 25 199–214. 10.2752/175303712X13316289505422

[B145] Scott J. P. Fuller J. L. (1965). *Genetics and the Social Behaviour of the Dog.* Chicago, IL: The University of Chicago Press.

[B146] Serpell J. A. (1986). *In the Company of Animals.* Oxford: Basil Blackwell.

[B147] Serpell J. A. Duffy D. L. Jagoe J. A. (2017). “Becoming a dog: Early experience and behaviour,” in *The Domestic Dog: Its Evolution, Behavior and Interactions with People*, 2nd Edn, ed. Serpell J. A. (Cambridge: Cambridge University Press), 93–117. 10.1097/01.aud.0000240596.56622.0c

[B148] Sharpley C. Veronese N. Smith L. López-Sánchez G. F. Bitsika V. Demurtas J. et al. (2020). Pet ownership and symptoms of depression: A prospective study of older adults. *J. Affect. Disord.* 264 35–39. 10.1016/j.jad.2019.11.134 31846900

[B149] Shaver P. R. Collins N. Clark C. L. (2014). “Attachment styles and internal working models of self and relationship partners,” in *Knowledge Structures in Close Relationships*, eds Fletcher G. J. O. Fitness J. (London: Psychology Press), 25–61.

[B150] Shipman P. (2015). *The Invaders. How Humans and Their Dogs Drove Neanderthals to Extinction.* Cambridge, MA: Belknap Press.

[B151] Singer T. (2006). The neuronal basis and ontogeny of empathy and mind reading: Review of literature and implications for future research. *Neurosci. Biobehav. Rev.* 30 855–863. 10.1016/j.neubiorev.2006.06.011 16904182

[B152] Sluzki C. E. (2010). The pathway between conflict and reconciliation: Coexistence as an evolutionary process. *Transcult. Psychiatry* 47 55–69. 10.1177/1363461510362650 20511252

[B153] Solomon J. Beetz A. Schöberl I. Gee N. Kotrschal K. (2019). Attachment security in companion dogs: Adaptation of Ainsworth’s strange situation and classification procedures to dogs and their human caregivers. *Attach. Hum. Dev.* 21 389–417. 10.1080/14616734.2018.1517812 30246604 PMC6532729

[B154] Stibel J. M. (2021). Decreases in brain size and encephalization in anatomically modern humans. *Brain Behav. Evol.* 96 64–77. 10.1159/000519504 34718234

[B155] Striedter G. F. Northcutt R. G. (2020). *Brains through Time. A Natural History of Vertebrates.* New York, NY: Oxford University Press.

[B156] Tabak B. A. Leng G. Szeto A. Parker K. J. Verbalis J. G. Ziegler T. E. et al. (2023). Advances in human oxytocin measurement: Challenges and proposed solutions. *Mol. Psychiatry* 28 127–140. 10.1038/s41380-022-01719-z 35999276 PMC9812775

[B157] Tarazona A. M. Ceballos M. C. Broom D. M. (2020). Human relationships with domestic and other animals: One health, one welfare, one biology. *Animals* 10:43. 10.3390/ani10010043 31878310 PMC7022888

[B158] Theofilou P. (2013). Quality of life: Definition and measurement. *Eur. J. Psychol.* 9 150–162. 10.5964/ejop.v9i1.337

[B159] Thornhill R. (2003). “Darwinian aesthetics informs traditional aesthetics,” in *Evolutionary Aesthetics*, eds Voland E. Grammer K. (Berlin: Springer), 9–35.

[B160] Tinbergen N. (1951). *The Study of Instinct.* Oxford: Oxford University Press.

[B161] Tinbergen N. (1963). On aims and methods of ethology. *Z. Tierpsychol.* 20 410–433. 10.1111/j.1439-0310.1963.tb01161.x

[B162] Topal J. Byrne R. W. Miklosi A. Csanyi V. (2006). Reproducing human actions and action sequences: “Do as I Do!” in a dog. *Anim.Cogn.* 9 355–367. 10.1007/s10071-006-0051-6 17024511

[B163] Tóth S. Kinczel A. Lengyel A. Pálinkás R. Molnár A. Kiss A. L. et al. (2023). The role of dogs in maintaining health and quality of life. *Geosport Soc.* 19 76–84. 10.30892/gss.1904-098

[B164] Tsuboi M. Husby A. Kotrschal A. Hayward A. Buechel S. D. Zidar J. et al. (2014). Comparative support for the expensive tissue hypothesis: Big brains are correlated with smaller gut and greater parental investment in Lake Tanganyika cichlids. *Evolution* 69 190–200. 10.1111/evo.12556 25346264 PMC4312921

[B165] Urquiza-Haas E. G. Kotrschal K. (2015). The mind behind anthropomorphic thinking: Attribution of mental states to other species. *Anim. Behav.* 109 167–176. 10.1016/j.anbehav.2015.08.011

[B166] Urquiza-Haas E. G. Kotrschal K. (2022). Human-animal similarity and the imageability of mental state concepts for mentalizing animals. *J. Cogn. Cult.* 22 220–245. 10.1163/15685373-12340133

[B167] Valstad M. Alvares G. A. Egknud M. Matziorinis A. M. Andreassen O. A. Westlye L. T. et al. (2017). The correlation between central and peripheral oxytocin concentrations: A systematic review and meta-analysis. *Neurosci. Biobehav. Rev.* 78 117–124. 10.1016/j.neubiorev.2017.04.017 28442403

[B168] van Hooff J. A. Aureli F. (2014). “Social homeostasis and the regulation of emotion,” in *Emotions*, eds van Goozen T. H. M. von de Poll N. E. Sergeant J. A. (London: Psychology Press), 197–217.

[B169] van IJzendoorn M. H. Dijkstra J. Bus A. G. (1995). Attachment, intelligence, and language: A meta-analysis. *Soc. Dev.* 4 115–128. 10.1111/j.1467-9507.1995.tb00055.x

[B170] Vasconcellos A. S. Virányi Z. Range F. Ades C. Scheidegger J. K. Möstl E. et al. (2016). Training reduces stress in human-socialised wolves to the same degree as in dogs. *PLoS One* 11:e0162389. 10.1371/journal.pone.0162389 27611784 PMC5017772

[B171] von Bartheld C. S. Bahney J. Herculano-Houzel S. (2016). The search for true numbers of neurons and glial cells in the human brain: A review of 150 years of cell counting. *J. Comp. Neurol.* 524 3865–3895. 10.1002/cne.24040 27187682 PMC5063692

[B172] von Uexküll J. (1909). *Umwelt und Innenwelt der Tiere.* Berlin: Springer.

[B173] Wagner C. Grob C. Hediger K. (2022). Specific and non-specific factors of animal-assisted interventions considered in research: A systematic review. *Front. Psychol.* 13:931347. 10.3389/fpsyg.2022.931347 35837630 PMC9274084

[B174] Wallis L. J. Range F. Müller C. A. Serisier S. Huber L. Zsó V. (2014). Lifespan development of attentiveness in domestic dogs: Drawing parallels with humans. *Front. Psychol.* 5:71. 10.3389/fpsyg.2014.00071 24570668 PMC3916763

[B175] Wascher C. A. F. Scheiber I. B. R. Kotrschal K. (2008). Heart rate modulation in bystanding geese watching social and non-social events. *Proc. R. Soc. B Biol. Sci.* 275 1653–1659. 10.1098/rspb.2008.0146 18430645 PMC2602813

[B176] Wedl M. Kotrschal K. (2009). Social and Individual components of animal contact in preschool children. *Anthrozoös* 22 383–396. 10.2752/089279309X12538695316220

[B177] Wheeler E. A. Faulkner M. E. (2015). The “pet effect”: Physiological calming in the presence of canines. *Soc. Anim.* 23 425–438. 10.1163/15685306-12341374

[B178] Wilkins A. S. (2020). A striking example of developmental bias in an evolutionary process: The “domestication syndrome”. *Evol. Dev.* 22 143–153. 10.1111/ede.12319 31545016

[B179] Wilkins A. S. Wrangham R. W. Fitch W. T. (2014). The “Domestication syndrome” in mammals: A unified explanation based on neural crest cell behavior and genetics. *Genetics* 197 795–808. 10.1534/genetics.114.165423 25024034 PMC4096361

[B180] Wilson E. O. (1984). *Biophilia.* Cambridge, MA: Harvard University Press.

[B181] Woodard K. Pollak S. D. (2020). Is there evidence for sensitive periods in emotional development? *Curr. Opin. Behav. Sci.* 36 1–6. 10.1016/j.cobeha.2020.05.004 32905224 PMC7467294

[B182] Wright H. Hall S. Hames A. Hardiman J. Mills R. Paws Project Team et al. (2015). Pet dogs improve family functioning and reduce anxiety in children with autism spectrum disorder. *Anthrozoös* 28 611–624. 10.1080/08927936.2015.1070003

[B183] Zentall T. R. (2023). Comparative cognition research demonstrates the similarity between humans and other animals. *Animals* 13:1165. 10.3390/ani13071165 37048420 PMC10093641

[B184] Zhang X. Li J. Xie F. Chen X. Xu W. Hudson N. W. (2022). The relationship between adult attachment and mental health: A meta-analysis. *J. Pers. Soc. Psychol.* 123 1089–1137. 10.1037/pspp0000437 36201836

